# The impact of a non-restrictive Antimicrobial Stewardship Program in the emergency department of a secondary-level Italian hospital

**DOI:** 10.1007/s11739-023-03418-1

**Published:** 2023-09-12

**Authors:** Caterina Monari, Lorenzo Onorato, Enrico Allegorico, Valentina Minerva, Margherita Macera, Giorgio Bosso, Federica Calò, Antonio Pagano, Teresa Russo, Gennaro Sansone, Marina D’Isanto, Antonio Casciotta, Monica Vanni, Fabio Giuliano Numis, Nicola Coppola

**Affiliations:** 1 Infectious Diseases Unit, Section of Infectious Diseases, Department of Mental Health and Public Medicine, University of Campania Luigi Vanvitelli, Via L. Armanni 5, 80131 Naples, Italy; 2 Department of Emergency and Critical Care, “Santa Maria Delle Grazie Hospital”, Pozzuoli, Italy; 3 Microbiology Unit, “Santa Maria Delle Grazie Hospital”, Pozzuoli, Italy; 4 Department of Pharmacology, “Santa Maria Delle Grazie Hospital, Pozzuoli, Italy; 5 ASL NAPOLI 2 Nord, Pozzuoli, Italy

**Keywords:** DDI, MDR, Emergency department, Antimicrobial stewardship, Audit

## Abstract

Evidence supporting the effectiveness of Antimicrobial Stewardship (AMS) Programs in the emergency department (ED) setting is limited. We conducted a prospective cohort study to assess the efficacy of an AMS program in an ED and a short-stay observation unit. The intervention included periodic prospective audits (twice a week), conducted by four infectious disease consultants. Primary outcomes included the difference in the hospital mortality rate, antibiotic consumption, and the incidence of bloodstream infections (BSI) caused by multidrug resistant (MDR) bacteria, before March 2020–February 2021 and after March 2021–February 2022 when the program was implemented. Interrupted time-series analysis was performed to assess the effect of our program. During the 12-month program, we performed 152 audits and evaluated 366 antibiotic therapies out of a total of 853 patients admitted. In the intervention period, we observed a non-statistically significant decrease in total antibiotic consumption, with a change in level of − 31.2 defined daily dose/100 patient-days (PD) (*p* = 0.71). Likewise, we found no significant variations in the rate of BSI due to MDR Gram-positive (CT − 0.02 events/PD, *p* = 0.84), MDR Gram-negative bacteria (CT 0.08, *p* = 0.71), or *Candida* spp. (CT 0.008, *p* = 0.86). Conversely, we found a significant decrease in the mortality rate between the pre- and post-intervention periods (− 1.98 deaths/100 PD, CI − 3.9 to − 0.007, *p* = 0.049). The Antibiotic Stewardship Program in the ED was associated with a significant decrease in the mortality rate. More high-quality studies are needed to determine the most effective ASP strategies in this unique setting.

**Supplementary Information:**

The online version contains supplementary material available at 10.1007/s11739-023-03418-1.

## Introduction

Antimicrobial resistance (AMR) is one of the main global health threats of the twenty-first century, significantly increasing the overall hospital mortality rate, the need for intensive care units (ICUs), the length of stay, and the healthcare costs [1].

A recently published predictive statistical model by Murray et al*. *[2], providing the first assessment of the global burden of AMR, estimated 4.95 million (3.62–6.57) deaths globally attributable to bacterial AMR in 2019. The six leading pathogens associated with antibiotic resistance were *Escherichia coli*, followed by *Staphylococcus aureus, Klebsiella pneumoniae, Streptococcus pneumoniae, Acinetobacter baumannii*, and *Pseudomonas aeruginosa *[2]. Moreover, it is currently estimated that, if no appropriate measures are taken, AMR will cost approximately 10 million lives and 10 trillion US dollars per year by 2050 [3].

To tackle the emergence of resistance, many regulatory authorities and scientific societies, such as the World Health Organization (WHO) and the European Commission and the Center for Disease Control and Prevention (ECDC), have endorsed guidelines that recommend the implementation of “Antibiotic Stewardship Programs” (ASP), to improve the appropriateness of antibiotic prescriptions, including the indication, the choice of molecules, the route of administration, and the duration of therapy [1, 4, 5].

Several studies have demonstrated a clear benefit from the implementation of stewardship programs on antibiotic consumption, selection of resistance, hospital mortality, and costs [6–10]. However, emergency departments (EDs) are a very particular setting, since they represent the interface between the community and hospitals, serving as a gateway of entry into the hospital [11]. Moreover, they are often overcrowded, with a high turnover of patients, the decision-making process is rapid, and antimicrobial prescriptions are usually empiric due to the lack of microbiological results [12, 13]. However, there is still a lack of literature regarding ASPs in the EDs [13, 14].

The aim of the present study was to assess the impact of a non-restrictive educational Antimicrobial Stewardship Program on the appropriateness of antimicrobial prescription in an ED of a secondary-level hospital in the Campania Region in southern Italy.

## Methods

### Study design and setting

We conducted a prospective cohort study with an interrupted time-series analysis in an ED and a short-stay observation unit of a secondary-level non-teaching hospital in Pozzuoli (Santa Maria delle Grazie Hospital), in the Campania Region in southern Italy. Santa Maria delle Grazie Hospital is located in Pozzuoli, about 20 km from the AOU L. Vanvitelli in Naples. The hospital includes six beds in the emergency department and ten beds in the short-stay observation unit. The hospital does not have an Infectious Disease Unit or an ID consultant, and has not yet implemented an Infection Prevention and Control (IPC) program. The study period was from March 2020 to March 2022 and was designed in accordance with the guidelines of the ORION statement [15].

### Antimicrobial Stewardship Program

In March 2021, the Azienda Ospedaliera Universitaria of the University of Campania Luigi Vanvitelli started a 1-year Antimicrobial Stewardship Program in the ED of the Hospital Santa Maria delle Grazie of Pozzuoli, to improve the appropriateness of antibiotic prescription. Before the intervention, there were no restriction rules on antibiotic prescriptions.

We identified a multidisciplinary team, including four ID consultants, ED doctors, microbiologists, pharmacists, a statistician, and the Health Department director. ID consultants performed audits twice a week at regular intervals, every Monday and Thursday. During the audits, they evaluated all patients on antibiotic treatment, giving recommendations on indication, spectrum of action, choice of molecules, route of administration, and length of antibiotic therapies. Moreover, they were involved in writing and sharing protocols, i.e., for adequate collection of blood cultures and other microbiological samples and on empirical treatment of urinary tract infections, based on national and international recommendations. Lastly, during the audits, they evaluated the rate of adherence to these protocols.

Microbiologists provided updated reports on the susceptibility profiles of specific pathogens; pharmacists provided reports on antibiotic consumption and the related costs of the specific unit. Lastly, the statistician provided information on in-hospital mortality and length of hospital stay.

The pre-intervention phase included the period from March 2020 to February 2021, while the intervention period included the months from March 2021 to February 2022. Type and severity of infections, defined according to the Sepsis-3 definitions [16], as well as causative agents were collected for all patients who were admitted to the emergency department and emergency medicine unit during the two periods and received antimicrobial treatment.

### Outcomes

We defined primary and secondary outcomes. Primary outcomes included the in-hospital mortality rate expressed as deaths/100 PD, the difference in antibiotic consumption, expressed as defined daily dose (DDD) per 100 patient-days (PD) according to WHO definitions [17], and the frequency of bloodstream infections (BSI) caused by multidrug resistant organisms (MDROs), expressed as events/100 PD, before and after the implementation of the program. The MDR categorization was applied according to the European Centre for Disease Control (ECDC) definitions [18]. Secondary outcomes included the mean length of hospital stay and antibiotic costs, expressed as euros/100 PD. Finally, we compared the mean diagnosis-related group (DRG) weight, as a parameter of clinical complexity of patients between the two periods. All variables were collected and analyzed monthly. The outcomes recorded in the intervention period were compared with those recorded in the pre-intervention period.

### Statistical analysis

Continuous variables were reported as mean and standard deviation (SD) if normally distributed or as median and interquartile range (IQR) if not normally distributed, and categorical variables as absolute and relative frequencies. For continuous variables, differences were evaluated by the Student's *t* test; categorical variables were compared using the Chi-squared test, using exact procedures if needed. Interrupted time-series analysis (ITSA) [19] was performed to assess the effect of our AS program on mortality, DDDs, costs, and on the frequency of MDRO bloodstream infections. Differences were considered statistically significant at *p* < 0.05 (two-tailed tests). Analyses were performed by SPSS 23.0 (IBM, Armonk, NY, USA).

### Ethics approval

The study was approved by the Ethics Committee of the University of Campania Luigi Vanvitelli, Naples (n°17539i/2022). All procedures performed in this study were in accordance with the ethics standards of the institutional and/or national research committee and with the 1964 Helsinki declaration and its later amendments or comparable ethics standards.

## Results

### Audits and characteristics of patients

During the 12-month ASP, we performed 152 audits and evaluated 366 antibiotic therapies out of a total of 853 patients admitted. The type and the severity of infections in patients admitted to the emergency department during the pre- and post-intervention phase are displayed in Supplementary Table 1. The prevalence of the different type of infections was similar between the two periods, with the exception of urinary tract infections, that were more common in the post-intervention phase (23.9 vs. 30.1%, *p* = 0.04); regarding the severity of patients, a higher rate of subjects with sepsis were admitted during the post-intervention phase (26.9 vs. 37.4%, *p* < 0.001). The distribution of microorganisms isolated from blood cultures, urine cultures, and respiratory samples was comparable during the two periods, as shown in Supplementary Table 2.

The antimicrobial treatment was deemed appropriate in 60.2% of cases during the first trimester of intervention and in 70.8% during the last trimester.

### Hospital mortality

The variation in hospital mortality is shown in Table 1. In the intervention period, from March 2021 to February 2022, we found a significant decrease in the mortality rate compared to the pre-intervention period (− 1.98 deaths/100 PD, CI − 3.9 to − 0.007, *p* = 0.049).

**Table 1:** Effect of the AMS program on mean length of hospital stay and overall in-hospital mortality

	Parameter evaluated	Effect estimate	LCI	UCI	*p* value
Mortality	Change in level	− 1.98	− 3.95	− 0.01	0.05
	Change in trend	0.09	− 0.24	0.42	0.56
Mean length of hospital stay	Change in level	1.34	− 2.70	5.38	0.49
	Change in trend	0.001	− 0.71	0.72	0.99

*LCI* lower confidence interval, *UCI* upper confidence interval

### Antibiotic consumption

The variations in antibiotic consumption are shown in Table 2. We observed a non-statistically significant reduction in total antibiotic consumption during the two periods, with a change in level (CL) of − 31.2 DDD/100 PD (95% CI − 207.4 to 145.1, *p* = 0.71) (Table 2); in particular, a non-significant decrease was observed in piperacillin/tazobactam (change in trend, CT − 0.7, CI − 4.0 to 2.7 *p* = 0.67) and fluoroquinolones (CT − 1.2, CI − 4.6 to 2.3, *p* = 0.49). Moreover, we observed non-statistically significant variations in the use of carbapenems (CT 0.9, CI − 1.02 to 2.76, *p* = 0.35), third- and fourth-generation cephalosporins (CT 3.7, CI − 4.5 to 12.0, *p* = 0.36), and inhibitor-protected aminopenicillins (CT 3.28, − 3.7 to 10.3, *p* = 0.34).

**Table 2:** Effect of the AMS program on antibiotic consumption and costs

Antibiotic	Parameter evaluated	Effect estimate	LCI	UCI	*p* value
All antibiotics	Change in level	− 31.18	− 207.43	145.06	0.71
	Change in trend	6.71	− 18.43	31.86	0.58
Carbapenems	Change in level	5.57	− 8.01	19.14	0.40
	Change in trend	0.87	− 1.02	2.76	0.35
III/IV generation cephalosporins	Change in level	16.16	− 40.03	72.35	0.55
	Change in trend	3.74	− 4.52	12.01	0.36
Fluoroquinolones	Change in level	11.19	− 12.11	34.50	0.33
	Change in trend	− 1.16	− 4.61	2.29	0.49
Piperacillin/ tazobactam	Change in level	− 6.64	− 31	17.73	0.58
	Change in trend	− 0.68	− 4.03	2.67	0.67
Aminopenicillins/ BLI	Change in level	1.44	− 48.44	51.33	0.95
	Change in trend	3.28	− 3.72	10.28	0.34
Macrolides	Change in level	− 36.96	− 107.60	33.68	0.29
	Change in trend	− 0.86	− 11.68	9.97	0.87
Vancomycin	Change in level	13.24	1.84	24.63	0.03
	Change in trend	− 1.83	− 3.50	− 0.15	0.03
Daptomycin	Change in level	− 3.17	− 11.57	5.23	0.44
	Change in trend	0.20	− 1.01	1.40	0.74
Linezolid	Change in level	2.45	− 16.21	21.11	0.79
	Change in trend	− 0.51	− 3.19	2.16	0.69
Tigecycline	Change in level	2.17	− 3.97	8.32	0.47
	Change in trend	− 0.45	− 1.31	0.40	0.29
Echinocandins	Change in level	8.08	3.06	13.11	< 0.001
	Change in trend	− 0.55	− 1.26	0.15	0.12
Azoles	Change in level	2.23	− 12.08	16.54	0.75
	Change in trend	− 0.41	− 2.41	1.59	0.67
Aminoglycosides	Change in level	7.40	− 19.68	34.49	0.58
	Change in trend	− 0.12	− 4.19	3.96	0.95
Metronidazole	Change in level	5.36	− 7.14	17.86	0.38
	Change in trend	− 0.82	− 2.61	0.97	0.35
Cotrimoxazole	Change in level	11.21	− 8.98	31.41	0.26
	Change in trend	1.61	− 1.27	4.49	0.26
Cefazolin	Change in level	3.37	− 5.55	12.30	0.44
	Change in trend	− 0.78	− 2.01	0.45	0.2
Ceftaroline	Change in level	− 1.91	− 9.28	5.46	0.59
	Change in trend	0.35	− 0.69	1.40	0.49
Costs	Change in level	1580.8	91.2	3070.5	0.039
	Change in trend	35.6	− 135.6	136.9	0.71

*LCI* lower confidence interval, *UCI* upper confidence interval, *BLI* beta-lactamase inhibitors

### Bloodstream infection rate

The effect of the AMS program on the rate of BSI due to MDR pathogens is presented in Table 3. We found no significant variations in the rate of bloodstream infections (BSI) due to MDR Gram-positive (CT − 0.02, CI − 0.2 to 0.16, *p* = 0.84) or MDR Gram-negative bacteria (CT 0.08, CI − 0.37 to 0.53, *p* = 0.71), or *Candida* spp. (CT 0.008, CI − 0.08 to 0.1 *p* = 0.86), as well as in the overall incidence rate of BSI due to Gram-positive (CT: − 0.56, CI − 1.3 to 0.18, *p* = 0.13) and Gram-negative bacteria (CT − 0.02, CI − 0.87 to 0.83, *p* = 0.96).

**Table 3:** Effect of the AMS program on the rate of bloodstream infections

Micro-organisms	Parameter evaluated	Effect estimate	LCI	UCI	*p* value
Gram-negative BSI	Change in level	2.87	− 1.53	7.28	0.18
	Change in trend	− 0.02	− 0.87	0.83	0.96
MDR Gram-negative BSI	Change in level	1.96	− 0.41	4.33	0.10
	Change in trend	0.08	− 0.37	0.53	0.71
Gram-positive BSI	Change in level	2.77	− 1.05	6.58	0.14
	Change in trend	− 0.57	− 1.33	0.18	0.13
MDR Gram-positive BSI	Change in level	0.53	− 0.40	1.47	0.25
	Change in trend	− 0.02	− 0.20	0.16	0.84
Candidemia	Change in level	0.74	0.26	1.22	< 0.001
	Change in trend	0.01	− 0.08	0.10	0.86

*LCI* lower confidence interval, *UCI* upper confidence interval, *BSI* bloodstream infections, *MDR* multidrug resistant

### Secondary outcomes: mean length of hospital stay, antibiotic costs, and mean DRG weight

No significant difference was observed in the mean length of hospital stay (CL: 1.3 days, CI − 2.7 to 5.4, *p* = 0.49) during the study periods (Table 1). Regarding the costs, we observed a non-significant change in trend (35.6 euros/100PD/month, CI − 165.6 to 263.9, *p* = 0.71), with a significant increase in level (1580, CI 91.3 to 3070.5, *p* = 0.039) (Table 2). Finally, similar mean DRG weights were found between the two periods (2.4 ± 0.4 vs. 2.5 ± 0.3, *p* = 0.71).

## Discussion

In the present study, we reported the effects of the implementation of an ASP in an emergency department and a short-stay observation unit of a secondary-level hospital in the Campania region. We performed 152 audits and evaluated 366 antibiotic therapies: we observed a significant decrease in the mortality rate, while a non-statistically significant variation in other primary outcomes, i.e*.*, total antimicrobial consumption and incidence of bloodstream infections due to MDROs, as well as secondary outcomes, i.e., antibiotic expense and length of stay.

The reduction in hospital mortality, despite the higher rate of patients presenting with sepsis in the post-intervention phase, is an encouraging result, and in our opinion was mainly driven by the increase in the appropriateness of antimicrobial prescribing during the intervention period. As stated, protocols of empirical antimicrobial therapy based on local resistance rates and in accordance with international guidelines were compiled and shared with the colleagues of the emergency department. An indirect demonstration comes from the data on antibiotic appropriateness: although data on the appropriateness of antimicrobial prescriptions are unavailable for the pre-intervention period, an increase in rates of treatments deemed appropriate was registered from the first to the last trimester of intervention. Our results are consistent with several data present in the literature. A meta-analysis [20] including 37 observational studies assessing the impact of the adherence to guidelines on mortality demonstrated that the group of patients receiving antimicrobial treatment according to guidelines presented a significantly lower risk of death (RR 0.65, 95% CI 0.54–0.80, *p* < 0.0001). Focusing our attention on the emergency department, a retrospective study with propensity-matched analysis demonstrated that receiving an antibiotic prescription discordant with the guidelines was independently associated with 30-day mortality (hazard ratio 1.43, 95% CI 1.05–1.93) among a cohort of 630 patients admitted to the ED for severe pneumonia [21]. Moreover, many studies have demonstrated that bedside ID consultations can reduce mortality in specific clinical conditions, including *S. aureus *[22–24], *Enterococcus *spp. [25], and *Candida* spp. bloodstream infections [26]. A retrospective multicenter study enrolling 36,868 patients with *S. aureus* BSI from 2003 to 2014 demonstrated that subjects receiving an ID consultation presented a significant reduction in 30-day mortality [22]. Similar results were observed in a retrospective cohort study conducted among 1,691 US patients with Candida bloodstream infection [26].

The results regarding other primary outcomes are slightly in contrast with several studies published in the literature, which have demonstrated a clear benefit from the implementation of stewardship programs on antibiotic consumption and costs, and selection of resistance, in other settings [7, 9]. Several systematic reviews and meta-analyses have demonstrated significant decreases in antimicrobial consumption and costs, especially in the critical care setting [9, 27]. A network meta-analysis [28], including 42 publications, demonstrated that ASP can lead to a statistically significant reduction in the incidence of infections or colonization by MDR Gram-negative bacteria in adults admitted to ICUs. However, EDs are very particular, since they are where antibiotics are first prescribed [12, 29, 30]. Moreover, EDs are often overcrowded, with a high turnover of patients, the decision-making process is rapid, and antimicrobial prescriptions are usually empiric due to the lack of microbiological results [12, 13].

As early as 2013, May et al. shifted the attention to EDs, underscoring the need for Antimicrobial Stewardship Programs in these difficult settings [14]. We should point out that several peculiar aspects differentiate the Antimicrobial Stewardship approach in EDs compared to other in-hospital settings, as stated by May et al*. *[12]. First, the AS approach in EDs should focus on the clinical diagnosis, also through rapid diagnostic methods. Second, the AS should be centered on empirical therapy, focusing on the spectrum, route of administration, timing, and dosing interval. The start of an empirical therapy has a major impact on the next step since therapy is often continued by colleagues in both in-hospital and outpatient settings. In addition, the AS should emphasize the importance of collecting microbiological samples before starting antibiotic therapy, and following up patients who are discharged from hospital with an empirical treatment in an outpatient regimen. The latter point is, perhaps, the most challenging element discussed so far. Consequently, data obtained in other areas cannot be applied to the ED, and efficacy of ASP intervention in emergency departments is less clearly demonstrated compared to other hospital settings. A systematic review [31], including 43 studies on the clinical effect of ASPs in the ED, described favorable clinical outcomes in a limited number of studies; the most frequently reported benefits included an improvement in the delivery of care or a decrease in antibiotic prescription. Moreover, we should consider that most studies were judged as having an unclear or high risk of bias. Indeed, the methodology to evaluate the efficacy of ASP intervention in this setting is not well standardized. A systematic review [30], including 26 studies on Antimicrobial Stewardship Programs in EDs, described the use of heterogeneous indicators to monitor antimicrobial consumption, making it difficult to compare the results of the studies included.

Thus, while there is a growing body of literature showing that multifaceted interventions, the draft of clinical guidelines, and behavioral approaches on prescribers have an impact on antibiotic prescription in EDs, there is still a lack of high-quality studies. A prospective before–after study, conducted by Borde et al. in a non-trauma ED in Germany in 2015, aimed at reducing the use of third-generation cephalosporins and fluoroquinolones, since these antibiotics were associated with a high risk of selection of bacterial resistance [32]. The colleagues put in place a non-restrictive ASP based on guideline revision, education, intensified ID consultations and feedback, and described a significant decrease in cephalosporin use, but not in fluoroquinolone use nor overall antibiotic consumption [32]. A quasi-experimental prospective study published in 2020 [13] aimed at evaluating the impact of an ASP on antibiotic use and costs in an ED of a German hospital. The intervention included four phases that were articulated as follows: phase 1—collection of prospective epidemiological and clinical data, phase 2—development and dissemination of guidelines on empirical treatment, phase 3—prospective audit and feedback and an active infectious disease consultation service, and phase 4—random audit and periodical feedback. In the 4-year project, colleagues evaluated 42,886 patients. They reported a non-significant decrease in overall antibiotic use during phase 2 (CL − 31.12, *p* 0.861) and 3 (CL − 7.2, *p* 0.983). Moreover, they described a significant decrease in the mean yearly antibiotic costs from 691.5 euros/100 PD to 263 euros/100 PD in phase 4 (*p* < 0.001) and in the length of stay. The rate of *Clostridioides difficile* infections (CDI) decreased as well, especially during phase 2. The implementation of an ASP was not accompanied by an increase in the mortality rate, which remained stable over the whole study period (3.3% in phase 1 to 2.1% in phase 4, *p* 0.094).

An interesting explanation of our non-significant results is that colleagues working in the ED in Santa Maria delle Grazie Hospital were highly trained and updated on antimicrobials. In almost 65% of cases, antibiotic prescriptions were appropriate, compared to an extremely high frequency of inappropriate antimicrobial prescriptions in the EDs described in the literature, about 40–60% [12].

Our study has some limitations; in particular, the intervention was performed in only one ward, and a limited number of audits were conducted. Furthermore, we cannot be sure that the clinical characteristics of the subjects evaluated during the pre- and post-intervention periods were comparable, considering that the study was conducted in different phases of the COVID-19 pandemics. However, we observed similar types and etiologies of infections among the study periods, with a higher rate of patients presenting with sepsis during the post-intervention phase; moreover, the lack of a significant variation in the mean DRG weight among the study periods demonstrated a similar case mix of patients admitted, which strengthens the findings of our study.

In conclusion, antibiotics are frequently prescribed in EDs and short-stay observation units, and almost half of them are unnecessary or inappropriate [29, 33]. Since antimicrobial resistance is riding high at a global level, Antibiotic Stewardship Programs are needed in the emergency departments and should be tailored according to each setting. More high-level studies are needed to determine the most effective ASP strategies in this unique setting.

### Supplementary Information

Below is the link to the electronic supplementary material.

Supplementary file1 (DOCX 19 KB)

## Data Availability

Data can be requested to the corresponding author, N.C. (nicola.coppola@unicampania.it).
