# Supplementary material for: The impact of a non-restrictive Antimicrobial Stewardship Program in the emergency department of a secondary-level Italian hospital
Source: Intern Emerg Med. 2023 Sep 12;19(2):493–500. doi: 10.1007/s11739-023-03418-1 (PMC10954915; doi:10.1007/s11739-023-03418-1)
Supplement: Supplementary file 1 — Supplementary file1 (DOCX 19 KB) [file 11739_2023_3418_MOESM1_ESM.docx]

|  | Pre-intervention | Post-intervention | P value |
| --- | --- | --- | --- |
| N. Patients | 401 | 481 |  |
| Site of infection (n, %)   - Lower respiratory tract - Urinary tract - Skin and soft tissue - Intra-abdominal - Central nervous system - Infective endocarditis - Other | 146 (36.4)  96 (23.9)  34 (8.5)  108 (26.9)  6 (1.5)  5 (1.2)  6 (1.5) | 163 (33.9)  145 (30.1)  53 (11)  108 (22.4)  3 (0.6)  5 (1.0)  4 (0.8) | 0.43  **0.04**  0.21  0.12  0.19  0.74  0.35 |
| Severity (n, %)   - Sepsis - Septic shock | 108 (26.9)  59 (14.7) | 180 (37.4)  89 (18.5) | **<0.001**  0.13 |

|  | Pre-intervention | Post-intervention | P value |
| --- | --- | --- | --- |
| Blood cultures, n (%)   - E. coli - K. pneumoniae - P. aeruginosa - A. baumannii - Other Gram-negative - S. aureus - E. faecalis - E. faecium - Other Gram-positive - Candida spp. | 116  40 (34.5)  5 (4.3)  6 (5.2)  3 (2.6)  11 (9.4)  15 (12.9)  7 (6)  3 (2.6)  22 (19.9)  4 (3.4) | 132  36 (27.3)  15 (11.4)  6 (4.5)  6 (4.5)  15 (11.4)  24 (18.2)  10 (7.6)  3 (2.3)  14 (10.6)  3 (2.3) | 0.88 |
| Urine cultures, n (%)   - E. coli - K. pneumoniae - P. aeruginosa - A. baumannii - Other Gram-negative - Enterococcus spp - Other Gram-positive - Candida spp. | 77  39 (50.6)  12 (15.5)  8 (10.4)  3 (3.9)  10 (13)  1 (1.3)  1 (1.3)  3 (3.9) | 102  54 (52.9)  15 (14.7)  5 (4.9)  3 (2.9)  16 (15.7)  5 (4.9)  0 (0.0)  4 (3.9) | 0.43 |
| Respiratory samples, n (%)  - Enterobacterales  - Non-fermenting Gram-negative  - S. aureus  - Other Gram-positive | 18  9 (50)  3 (16.7)  5 (27.8)  1 (5.5) | 9  1 (11.1)  2 (22.2)  4 (44.5)  2 (22.2) | 0.21 |

**Supplementary table 1**. Type and severity of infections among patients admitted to the Emergency Department and Emergency Medicine Unit in pre- and post-intervention period.

**Supplementary table 2**. Clinical isolates of patients admitted to the Emergency Department and Emergency Medicine Unit in pre- and post-intervention period.
